# Thermoelectric Rectification and Amplification in Interacting Quantum-Dot Circuit-Quantum-Electrodynamics Systems

**DOI:** 10.3390/e25030498

**Published:** 2023-03-14

**Authors:** Jincheng Lu, Rongqian Wang, Chen Wang, Jian-Hua Jiang

**Affiliations:** 1 Jiangsu Key Laboratory of Micro and Nano Heat Fluid Flow Technology and Energy Application, School of Physical Science and Technology, Suzhou University of Science and Technology, Suzhou 215009, China; 2 Institute of Theoretical and Applied Physics, School of Physical Science and Technology & Collaborative Innovation Center of Suzhou Nano Science and Technology, Soochow University, Suzhou 215006, China; 3 Department of Physics, Zhejiang Normal University, Jinhua 321004, China

**Keywords:** thermoelectric effect, quantum transport, mesoscopic systems, thermoelectric rectification, thermal transistor

## Abstract

Thermoelectric rectification and amplification were investigated in an interacting quantum-dot circuit-quantum-electrodynamics system. By applying the Keldysh nonequilibrium Green’s function approach, we studied the elastic (energy-conserving) and inelastic (energy-nonconserving) transport through a cavity-coupled quantum dot under the voltage biases in a wide spectrum of electron–electron and electron–photon interactions. While significant charge and Peltier rectification effects were found for strong light–matter interactions, the dependence on electron–electron interaction could be nonmonotonic and dramatic. Electron–electron interaction-enhanced transport was found under certain resonance conditions. These nontrivial interaction effects were found in both linear and nonlinear transport regimes, which manifested in charge and thermal currents, rectification effects, and the linear thermal transistor effect.

## 1. Introduction

In recent years, there has been a lot of progress in probing and controlling hybrid quantum systems, which sit at the intersection of mesoscopic physics and quantum optics [1,2,3,4,5,6,7]. Some examples of such hybrid light–matter systems include circuit-quantum-electrodynamics (c-QED) systems [8,9,10,11,12,13,14] and quantum-dot (QD) c-QED systems [15,16,17,18]. One interesting kind of setup is quantum dots at a finite voltage bias, which has been integrated with superconducting microwave resonators, accomplishing sufficiently strong light–matter coupling [19,20,21,22]. Such QD cQED systems offer a rich platform for studying quantum transport [7] and nonequilibrium thermodynamics [3]. Experiments are versatile, tunable (with a broad range of parameters), and scalable. These QD c-QED setups are important from both a fundamental perspective (investigating correlations, transport, entanglement, and bosonic statistics) and the point of view of device applications (quantum microwave amplifiers and lasers in microwave regimes). Furthermore, from the perspective of devices, the focus and success until now have been related to realizing photon emitters [23], microwave amplifiers [24,25,26], and even single-atom lasers [27].

The manipulation and separation of electrical and heat currents at mesoscopic scales are important for high-performance thermoelectric quantum devices and other nanoscale machines [28,29,30,31,32,33,34,35,36,37,38,39,40,41,42,43,44,45,46]. One particularly attractive direction is to convert the electromagnetic energy from microwave quantum cavities into electrical work, or use such energy to control electron motion in mesoscopic systems. Although much excellent work has been devoted to studying the effects of electron–phonon [47,48], electron–photon [49], and electron–electron [50,51,52,53] interactions on thermoelectric transport, discussions of thermoelectric transistors and diodes in a strong coupling regime are still lacking. In this work, we investigated the inelastic thermoelectric transport in a single QD that was strongly coupled to photons residing inside a microwave cavity [54,55,56,57,58,59,60,61,62,63,64]. Strong or even ultra-strong couplings provide a great avenue for realizing novel quantum devices. Coulomb interactions may drive interesting strongly correlated electronic states such as density waves, magnetic order, and superconductivity [65]. By employing the nonequilibrium Green’s function approach [66,67], we studied the quantum thermoelectric transport through a single interacting quantum dot at a finite voltage bias coupled to a microwave field [68,69,70,71,72]. The present calculations were carried out for the Hubbard model with finite electron–electron interactions, as is appropriate for nanoscale-sized quantum dots [73]. We considered the electron–photon interactions beyond the predictions based on conventional second-order perturbation approximations [49,74]. We showed that due to the nonlinearity induced by electron–photon and electron–electron coupling, significant electric and rectification effects could be realized by tuning various parameters. We further showed that such a QD c-QED setup exhibited thermal transistor effects even in the linear regime without relying on negative differential thermal conductance.

The paper is organized as follows. In Section 2, we introduce the QD c-QED system. Section 3 and Section 4 are devoted to describing the nonequilibrium Green’s function method and calculating the elastic and inelastic currents in our system. Section 5 is devoted to studying the rectification effects in these devices, and we show that the rectification could be enhanced by suitably tuning the parameters of the QD c-QED system. In Section 6, we describe the thermoelectric coefficients in the linear response regime, and the elements of the Onsager relations are explained and investigated based on our QD cQED system. The section is devoted to demonstrating that these devices could act as a thermal transistors even in the linear response regime. We summarize our findings in Section 7, along with our future outlook.

## 2. Method and Approximation

**Figure 1:**
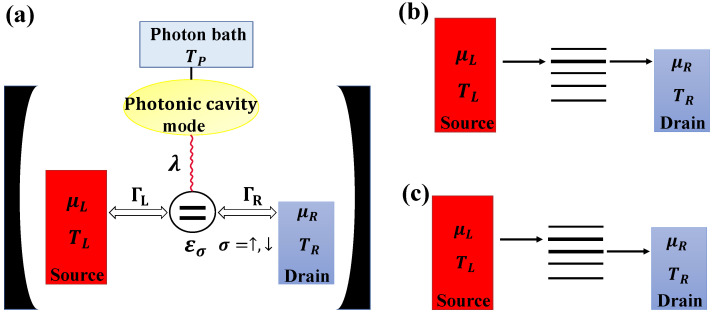
(**a**) A schematic representation of the QD c-QED system. The mesoscopic system was effectively housed in the microwave cavity. Wavy lines indicate the light–matter coupling λ. Tunneling rates between the quantum dot ($\varepsilon_{\sigma }$) and the electron leads ($\Gamma_{L}$, $\Gamma_{R}$) could be tuned via gate-controlled tunnel barriers. Electrons traveled from the source into the quantum dot and then hopped to the drain assisted by a photon from the photonic bath. (**b**) Illustration of possible elastic transport processes. (**c**) Illustration of possible photon-assisted inelastic transport processes.

We considered a single quantum dot connected to fermionic reservoirs (chemical potentials $\mu_{L}$ and $\mu_{R}$, $\Delta \mu =\mu_{L}-\mu_{R}$), as schematically depicted in Figure 1a.

The quantum dot could be modeled based on an InAs nanowire and was coupled to a photon mode, which was in turn coupled to a bath. We start with a microscopic description of the whole system:

(1) $$\hat{H}={\hat{H}}_{\mathrm{polaron}}+{\hat{H}}_{\mathrm{lead}}+{\hat{H}}_{\mathrm{dot}-\mathrm{lead}},$$

where

(2) $${\hat{H}}_{\mathrm{polaron}}={\hat{H}}_{\mathrm{dot}}+{\hat{H}}_{p}+{\hat{H}}_{e-p}+{\hat{H}}_{e-e},$$

with

(3a) $$\begin{array}{cc} \; & {\hat{H}}_{\mathrm{dot}}=\sum_{\sigma }\;\varepsilon_{\sigma }{\hat{d}}_{\sigma }^{†}\;{\hat{d}}_{\sigma }, \end{array}$$

(3b) $$\begin{array}{cc} \; & {\hat{H}}_{p}=\hbar \omega_{0}{\hat{a}}^{†}\hat{a}, \end{array}$$

(3c) $$\begin{array}{cc} \; & {\hat{H}}_{e-p}=\lambda \left(\hat{a}+{\hat{a}}^{†}\right)\sum_{\sigma }\;{\hat{d}}_{\sigma }^{†}\;{\hat{d}}_{\sigma }, \end{array}$$

(3d) $$\begin{array}{cc} \; & {\hat{H}}_{e-e}=U{\hat{d}}_{↑}^{†}\;{\hat{d}}_{↑}{\hat{d}}_{↓}^{†}\;{\hat{d}}_{↓}. \end{array}$$

In the first expression, ${\hat{d}}_{\sigma }^{†}$ and ${\hat{d}}_{\sigma }$ create and annihilate an electron of spin σ at the quantum-dot energy level $\varepsilon_{\sigma }$. In the second expression, ${\hat{a}}^{†}$ and $\hat{a}$ create and annihilate a photon with energy $\omega_{0}$. The third expression describes an electron–photon (e-p) interaction with strength λ. The fourth expression denotes an electron–electron (e-e) interaction with strength *U*. The Hubbard *U* term originates from the finite electron–electron repulsion in a single quantum dot, due to possible electron accumulation [50,51,52,53,75].

The Hamiltonians,

(4) $${\hat{H}}_{\mathrm{lead}}=\sum_{k\sigma \alpha }\varepsilon_{k\alpha }{\hat{d}}_{k\alpha \sigma }^{†}{\hat{d}}_{k\alpha \sigma },$$

(5) $${\hat{H}}_{\mathrm{dot}-\mathrm{lead}}=\sum_{k\sigma \alpha }V_{k\alpha }\left({\hat{d}}_{k\alpha \sigma }^{†}{\hat{d}}_{\sigma }+H.c.\right),$$

describe the electronic leads and the tunneling between the quantum dot and the leads, respectively.

## 3. Non-Perturbative Hybridized Dot Green’s Function

We introduced a cavity photon basis with displacements shifted by quantum-dot states through the e-p coupling [73,76]:

(6) $${|n\rangle }_{\nu }=\left[{\left({\hat{A}}_{\nu }^{†}\right)}^{n}/\sqrt{n!}\right]\exp\left(-g_{\nu }^{2}/2-g_{\nu }{\hat{a}}^{†}\right)|0\rangle ,$$

where ${\hat{A}}_{\nu }^{†}={\hat{a}}^{†}+g_{\nu }$ denotes the creator that generates a photon displaced from the original position; $g_{\nu }$ depends on the electronic state, i.e., $g_{0}=0$, $g_{\bar{\sigma }}=g_{\sigma }=g$, and $g_{\sigma \bar{\sigma }}=g_{\sigma }+g_{\bar{\sigma }}=2g$; and the photon excitation number is given by n=0,1,2… Therefore, with the help of the cavity photon basis, the solution to the eigenvalue problem is:

(7a) $$\begin{array}{cc} {}_{0}\langle 0,n|H_{\mathrm{polaron}}{|0,n\rangle }_{0} & =n\omega_{0}, \end{array}$$

(7b) $$\begin{array}{cc} {}_{\sigma }\langle \sigma ,n|H_{\mathrm{polaron}}{|\bar{\sigma },n\rangle }_{\sigma } & =n\omega_{0}+{\tilde{\varepsilon }}_{\sigma }, \end{array}$$

(7c) $$\begin{array}{cc} {}_{\bar{\sigma }}\langle \bar{\sigma },n|H_{\mathrm{polaron}}{|\bar{\sigma },n\rangle }_{\bar{\sigma }} & =n\omega_{0}+{\tilde{\varepsilon }}_{\bar{\sigma }}, \end{array}$$

(7d) $$\begin{array}{cc} {}_{\sigma \bar{\sigma }}\langle \sigma \bar{\sigma },n|H_{\mathrm{polaron}}{|\sigma \bar{\sigma },n\rangle }_{\sigma \bar{\sigma }} & =n\omega_{0}+{\tilde{\varepsilon }}_{\sigma \bar{\sigma }}, \end{array}$$

where ${\tilde{\varepsilon }}_{\sigma }=\varepsilon_{\sigma }-\omega_{0}g_{\sigma }^{2}$, ${\tilde{\varepsilon }}_{\bar{\sigma }}=\varepsilon_{\bar{\sigma }}-\omega_{0}g_{\bar{\sigma }}^{2}$ and ${\tilde{\varepsilon }}_{\sigma \bar{\sigma }}={\tilde{\varepsilon }}_{\sigma }+{\tilde{\varepsilon }}_{\bar{\sigma }}-\omega_{0}g_{\sigma }g_{\bar{\sigma }}$.

Obviously, ${|0,n\rangle }_{0}$, ${|\sigma ,n\rangle }_{\sigma }$, $|\bar{\sigma }{,n\rangle }_{\bar{\sigma }}$, $|\sigma \bar{\sigma }{,n\rangle }_{\sigma \bar{\sigma }}$ are four possible eigenstates, and $n\omega_{0}$, $n\omega_{0}+{\tilde{\varepsilon }}_{\sigma }$, $n\omega_{0}+{\tilde{\varepsilon }}_{\bar{\sigma }}$, $n\omega_{0}+{\tilde{\varepsilon }}_{\sigma \bar{\sigma }}$ are the corresponding possible eigenvalues. The negative term $2\omega_{0}g_{\sigma }g_{\bar{\sigma }}$ is evidence for the attractive interaction between the different electron states induced by electron–photon coupling.

Based on [73], we used the retarded (advanced) Green’s functions:

(8) $$\begin{array}{c} G_{0,\sigma }^{r(a)}\left(\omega \right)=\frac{1}{Z}\sum_{n,m=0}^{\infty }\left[\frac{e^{-\beta m\omega_{0}}+e^{-\beta (n\omega_{0}+{\tilde{\varepsilon }}_{\sigma })}}{\omega -\Delta_{mn}^{\left(1\right)}\pm i0^{+}}+\frac{e^{-\beta (m\omega_{0}+{\tilde{\varepsilon }}_{\bar{\sigma }})}+e^{-\beta (n\omega_{0}+{\tilde{\varepsilon }}_{\sigma \bar{\sigma }}+U)}}{\omega -\Delta_{mn}^{\left(2\right)}\pm i0^{+}}\right]D_{nm}^{2}\left(g_{\sigma }\right),\; \end{array}$$

where

$$\begin{array}{cc} \Delta_{nm}^{\left(1\right)} & =\left(n-m\right)\omega_{0}+{\tilde{\varepsilon }}_{\sigma },\\ \Delta_{nm}^{\left(2\right)} & =\left(n-m\right)\omega_{0}+\left({\tilde{\varepsilon }}_{\sigma }-2\omega_{0}g_{\sigma }g_{\bar{\sigma }}+U\right),\\ D_{nm}\left(g_{\sigma }\right) & =e^{-g_{\sigma }^{2}/2}\sum_{k=0}^{\min\{n,m\}}\frac{{\left(-1\right)}^{k}\sqrt{n!m!}g_{\sigma }^{n+m-2k}}{(n-k)!(m-k)!k!},\\ Z & =\left(1+N_{P}\right)\left(1+e^{-\beta {\tilde{\varepsilon }}_{\sigma }}+e^{-\beta {\tilde{\varepsilon }}_{\bar{\sigma }}}+e^{-\beta ({\tilde{\varepsilon }}_{\sigma \bar{\sigma }}+U)}\right). \end{array}$$

$N_{P}=1/\left(e^{\beta \omega_{0}}-1\right)$ denotes the Bose–Einstein distribution of the photon field, with the inverse temperature $\beta \equiv 1/k_{B}T_{P}$.

## 4. Inelastic and Elastic Currents

Using the nonequilibrium Green’s functions, we could study the quantum transport by calculating the charge current [77]

(9) $$I_{e}^{L}=e\frac{d}{dt}\langle \sum_{i}{\hat{d}}_{i}^{†}{\hat{d}}_{i}\rangle =e\int \frac{d\omega }{2\pi }I_{L}\left(\omega \right)$$

and the heat current

(10) $$I_{Q}^{L}=\frac{d}{dt}\langle \sum_{i}\left(\epsilon_{i}^{L}-\mu_{L}\right){\hat{d}}_{i}^{†}{\hat{d}}_{i}\rangle =\int \frac{d\omega }{2\pi }\left(\omega -\mu_{L}\right)I_{L}\left(\omega \right)$$

leaving electrode *L*. The Green’s function calculation yields

(11) $$\begin{array}{c} I_{L}\left(\omega \right)=-i\sum_{\sigma }\left(\Gamma_{L}\left(\omega \right)G_{tot,\sigma }^{<}\left(\omega \right)-f_{L,\sigma }\left(\omega \right)\left[G_{tot,\sigma }^{a}\left(\omega \right)-G_{tot,\sigma }^{r}\left(\omega \right)\right]\right), \end{array}$$

where the Green’s functions $G_{tot,\sigma }^{<}\left(\omega \right)$, $G_{tot,\sigma }^{a}\left(\omega \right)$, and $G_{tot,\sigma }^{r}\left(\omega \right)$ are the lesser, advanced, and retarded Green’s functions, respectively. Moreover, by using the Dyson equation and the Keldysh formula, we obtained the total retarded (advanced) Green’s function [77]:

(12) $$G_{tot,\sigma }^{<}\left(\omega \right)=G_{tot,\sigma }^{a}\left(\omega \right)\left[\Sigma_{P,\sigma }^{<}\left(\omega \right)+\Sigma_{1,\sigma }^{<}\left(\omega \right)\right]G_{tot,\sigma }^{r}\left(\omega \right),$$

where

(13) $$G_{tot,\sigma }^{r}\left(\omega \right)={\left[{\left(G_{1,\sigma }^{r}\left(\omega \right)\right)}^{-1}-\Sigma_{P,\sigma }^{r}\left(\omega \right)\right]}^{-1},$$

with

(14) $$G_{1,\sigma }^{r}\left(\omega \right)={\left[{\left(G_{0,\sigma }^{r}\left(\omega \right)\right)}^{-1}-\Sigma_{1,\sigma }^{r}\left(\omega \right)\right]}^{-1}.$$

As seen from the above equations, the self-energy on the dot included two contributions, $\Sigma_{1,\sigma }^{<}\left(\omega \right)$ and $\Sigma_{P,\sigma }^{<}\left(\omega \right)$. The first self-energy contribution $\Sigma_{1}$ originated from the electron–lead interactions, which more accurately should include the on-site electron–electron repulsion. Here, we focused on the sequential transport regime and considered the noninteracting electron dot self-energy as the lowest-order approximation. Therefore, $\Sigma_{1}$ was approximated as [66]

(15) $$\begin{array}{cc} \; & \Sigma_{1,\sigma }^{>}=-i\left[\Gamma_{L}\left(1-f_{L,\sigma }\right)+\Gamma_{R}\left(1-f_{R,\sigma }\right)\right], \end{array}$$

(16) $$\begin{array}{cc} \; & \Sigma_{1,\sigma }^{<}=i\left(\Gamma_{L}f_{L,\sigma }+\Gamma_{R}f_{R,\sigma }\right), \end{array}$$

(17) $$\begin{array}{cc} \; & \Sigma_{1,\sigma }^{r(a)}=\mp i\left(\Gamma_{L}+\Gamma_{R}\right)/2. \end{array}$$

Here, $f_{L(R),\sigma }={\left[e^{\left(\omega -\mu_{L(R),\sigma }\right)/k_{B}T_{L(R)}}+1\right]}^{-1}$ is the Fermi–Dirac distribution of the left (right) reservoir, $\mu_{L(R),\sigma }=\mu_{\sigma }\pm \Delta \mu /2$ is the chemical potential, and Δμ is the potential difference.

Moreover, there could be spin-dependent chemical potentials when the left and/or the right leads were ferromagnetic, which were then characterized as the spin chemical potential differences $\mu_{L,\sigma }-\mu_{L,\bar{\sigma }}=\delta \mu_{L}$ and $\mu_{R,\sigma }-\mu_{R,\bar{\sigma }}=\delta \mu_{R}$. $\Gamma_{L(R)}\equiv 2\pi \sum_{k}{\left|V_{kL(R)}\right|}^{2}\delta \left(\omega -\epsilon_{k\sigma }^{L(R)}\right)$ denotes the dot-lead coupling function, and e<0 is the electron charge.

To break the left–right reflection symmetry and induce efficient energy filtering, we set the tunnel coupling in the form of Lorentzian functions [28]:

(18) $$\Gamma_{L}\left(\omega \right)=\frac{\Gamma_{0}^{3}}{{\left(\omega -E_{l}\right)}^{2}+\Gamma_{0}^{2}},\;\;\;\;\Gamma_{R}\left(\omega \right)=\frac{\Gamma_{0}^{3}}{{\left(\omega -E_{r}\right)}^{2}+\Gamma_{0}^{2}}.$$

The second contribution to the self-energy resulted from the interaction with the photons, which in the leading order read [74]:

(19) $$\begin{array}{cc} \Sigma_{P,\sigma }^{r}\left(\omega \right)= & i\lambda^{2}\int \frac{d\omega^{\prime }}{2\pi }\left[\frac{\left(1+N_{P}\right)G_{1,\sigma }^{>}\left(\omega^{\prime }\right)-N_{P}G_{1,\sigma }^{<}\left(\omega^{\prime }\right)}{\omega -\omega_{0}-\omega^{\prime }+i0^{+}}+\frac{N_{P}G_{1,\sigma }^{>}\left(\omega^{\prime }\right)-\left(1+N_{P}\right)G_{1,\sigma }^{<}\left(\omega^{\prime }\right)}{\omega +\omega_{0}-\omega^{\prime }+i0^{+}}\right],\; \end{array}$$

and

(20) $$\begin{array}{c} \Sigma_{P,\sigma }^{<}\left(\omega \right)=\lambda^{2}\left[N_{P}G_{1,\sigma }^{<}\left(\omega -\omega_{0}\right)+\left(1+N_{P}\right)G_{1}^{<}\left(\omega +\omega_{0}\right)\right]. \end{array}$$

Inserting the expressions for the Green’s function $G_{tot,\sigma }$ into the above equation, one would find that $I_{L}$ could be written as a sum of two terms, the first arising from the elastic transitions of the transport electrons, and the other coming from the inelastic ones:

(21) $$\begin{array}{c} I_{L}\left(\omega \right)=I_{L}^{el}\left(\omega \right)+I_{L}^{inel}\left(\omega \right). \end{array}$$

The elastic-process contribution is described as

(22) $$\begin{array}{c} I_{L}^{\mathrm{el}}\left(\omega \right)=\sum_{\sigma }\left(\Gamma_{L}\left(\omega \right)G_{tot,\sigma }^{r}\left(\omega \right)\left[\Sigma_{l,\sigma }^{<}\left(\omega \right)+2f_{L,\sigma }\left(\omega \right)\Sigma_{l,\sigma }^{r}\left(\omega \right)\right]G_{tot,\sigma }^{a}\left(\omega \right)\right), \end{array}$$

while the inelastic contribution is given by

(23) $$\begin{array}{c} I_{L}^{\mathrm{inel}}\left(\omega \right)=\sum_{\sigma }\left(\Gamma_{L}\left(\omega \right)G_{1,\sigma }^{r}\left(\omega \right)\left[\Sigma_{P,\sigma }^{<}\left(\omega \right)+2f_{L,\sigma }\left(\omega \right)\Sigma_{P,\sigma }^{r}\left(\omega \right)\right]G_{1,\sigma }^{a}\left(\omega \right)\right). \end{array}$$

Thus, we could obtain the elastic and inelastic currents as follows:

(24a) $$\begin{array}{cc} \; & I_{e}^{L}{|}_{\mathrm{el}}=\frac{e}{\hbar }\int \frac{d\omega }{2\pi }\sum_{\sigma }\left(\Gamma_{L}\left(\omega \right)G_{tot,\sigma }^{r}\left(\omega \right)\left[\Sigma_{l,\sigma }^{<}\left(\omega \right)+2f_{L,\sigma }\left(\omega \right)\Sigma_{l,\sigma }^{r}\left(\omega \right)\right]G_{tot,\sigma }^{a}\left(\omega \right)\right), \end{array}$$

(24b) $$\begin{array}{cc} \; & I_{e}^{L}{|}_{\mathrm{inel}}=\frac{e}{\hbar }\int \frac{d\omega }{2\pi }\sum_{\sigma }\left(\Gamma_{L}\left(\omega \right)G_{1,\sigma }^{r}\left(\omega \right)\left[\Sigma_{P,\sigma }^{<}\left(\omega \right)+2f_{L,\sigma }\left(\omega \right)\Sigma_{P,\sigma }^{r}\left(\omega \right)\right]G_{1,\sigma }^{a}\left(\omega \right)\right). \end{array}$$

We also computed the heat current as follows:

(25a) $$\begin{array}{cc} \; & I_{Q}^{L}{|}_{\mathrm{el}}=\frac{1}{\hbar }\int \frac{d\omega }{2\pi }\sum_{\sigma }\left(\omega -\mu_{L,\sigma }\right)\left(\Gamma_{L}\left(\omega \right)G_{tot,\sigma }^{r}\left(\omega \right)\left[\Sigma_{l,\sigma }^{<}\left(\omega \right)+2f_{L,\sigma }\left(\omega \right)\Sigma_{l,\sigma }^{r}\left(\omega \right)\right]G_{tot,\sigma }^{a}\left(\omega \right)\right), \end{array}$$

(25b) $$\begin{array}{cc} \; & I_{Q}^{L}{|}_{\mathrm{inel}}=\frac{1}{\hbar }\int \frac{d\omega }{2\pi }\sum_{\sigma }\left(\omega -\mu_{L,\sigma }\right)\left(\Gamma_{L}\left(\omega \right)G_{1,\sigma }^{r}\left(\omega \right)\left[\Sigma_{P,\sigma }^{<}\left(\omega \right)+2f_{L,\sigma }\left(\omega \right)\Sigma_{P,\sigma }^{r}\left(\omega \right)\right]G_{1,\sigma }^{a}\left(\omega \right)\right). \end{array}$$

After some algebraic calculations, we found that the elastic charge and heat currents flowing out of the left lead were expressed as:

(26a) $$\begin{array}{cc} \; & I_{e}^{L}{|}_{\mathrm{el}}=\frac{e}{\hbar }\int \frac{d\omega }{2\pi }\sum_{\sigma }T_{el,\sigma }\left(\omega \right)\left[f_{L}\left(\omega \right)-f_{R}\left(\omega \right)\right], \end{array}$$

(26b) $$\begin{array}{cc} \; & I_{Q}^{L}{|}_{\mathrm{el}}=\frac{1}{\hbar }\int \frac{d\omega }{2\pi }\sum_{\sigma }\left(\omega -\mu_{L,\sigma }\right)T_{el,\sigma }\left(\omega \right)\left[f_{L}\left(\omega \right)-f_{R}\left(\omega \right)\right]. \end{array}$$

where $T_{\mathrm{el},\sigma }\left(\omega \right)=\Gamma_{L}\left(\omega \right)\Gamma_{R}\left(\omega \right){\left|G_{tot,\sigma }^{r}\left(\omega \right)\right|}^{2}$ is the transmission function for the elastic current.

For the charge and heat currents flowing out of the *R* lead, the same expressions held once L→R. Charge conservation implied that $I_{e}^{L}+I_{e}^{R}=0$ [33], while energy conservation required $I_{Q}^{L}+I_{Q}^{R}+I_{Q}^{P}+\mu_{L}I_{e}^{L}/e+\mu_{R}I_{e}^{R}/e=0$ [77]. The net charge current flowing from the left reservoir to the right reservoir was then

(27) $$\begin{array}{c} I_{e}=\frac{1}{2}\left(I_{e}^{R}-I_{e}^{L}\right). \end{array}$$

The heat current flowing into the photonic bath was

(28) $$\begin{array}{c} I_{Q}^{P}=-\left(I_{Q}^{L}+I_{Q}^{R}+\frac{\mu_{L}}{e}I_{e}^{L}+\frac{\mu_{R}}{e}I_{e}^{R}\right), \end{array}$$

and the net heat current exchanged between the *L* and *R* leads was

(29) $$I_{Q}=\frac{1}{2}\left(I_{Q}^{R}-I_{Q}^{L}\right).$$

Compared with [76], the perturbation theory (in λ) employed here well reproduced the quantitative features of the charge and heat transport. The inelastic current was treated in a perturbative way, and we included the normal ($O(\lambda^{2})$) and higher-order terms in noncrossing diagrams. However, we neglected the fourth-order ($\lambda^{4}$) and higher-order terms in the crossing diagrams [76]. The dot-lead coupling $\Gamma_{L}$ and $\Gamma_{R}$ were very small compared to all other energy scales. Our perturbation theory was valid for $\lambda <0.3\hbar \omega_{0}$, where the higher-order corrections were small $O(\frac{\lambda^{2}}{\hbar^{2}\omega_{0}^{2}})≲0.09\ll 1$. In this model, we assumed that the c-QED system had a temperature $k_{B}T_{P}$, which was justified in the linear-response regime or when the dot-lead couplings were much weaker than the light–matter interactions.

Unless otherwise stated, we set $\varepsilon_{\sigma }\equiv 0$, $\Gamma_{0}=0.05\hbar \omega_{0}$, and the temperatures of the electronic reservoir and photon bath were identical, i.e., $k_{B}T_{L}=k_{B}T_{R}=k_{B}T_{P}\equiv k_{B}T$ and $k_{B}T=0.1\hbar \omega_{0}$. We also did not consider the effect of spin on the QD QED system, and $\mu_{L/R,↑}=\mu_{L/R,↓}$, i.e., $\delta \mu_{L}=\delta \mu_{R}\equiv 0$.

**Figure 2:**
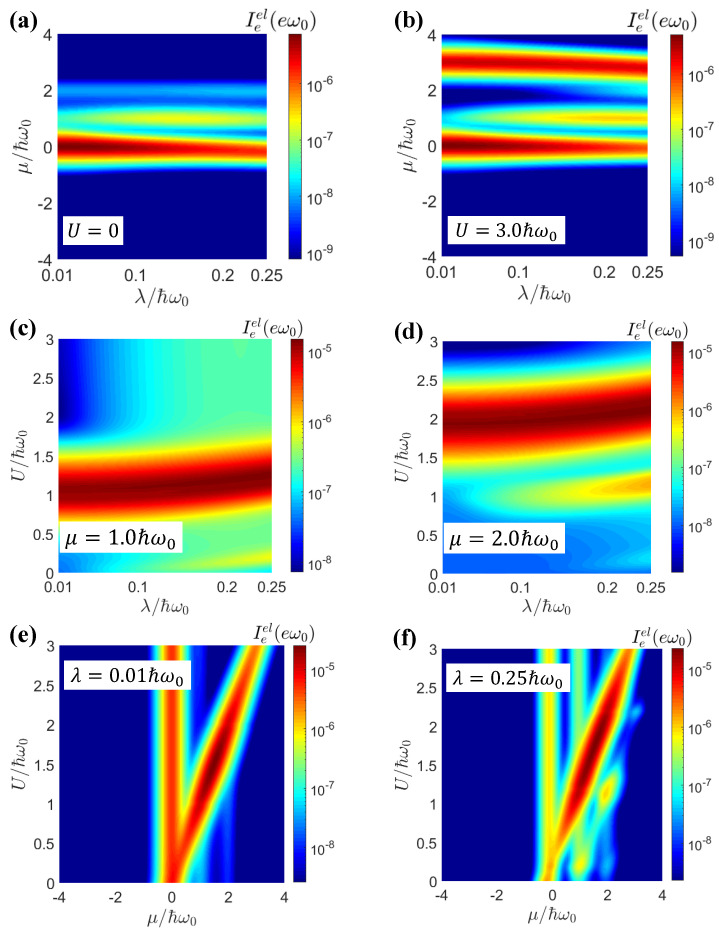
The elastic current $I_{e}^{\mathrm{el}}$ as a function of λ and μ for (**a**) U=0 and (**b**) $U=3.0\hbar \omega_{0}$. The elastic current $I_{e}^{\mathrm{el}}$ as a function of λ and *U* for (**c**) $\mu =1.0\hbar \omega_{0}$ (**d**) $\mu =2.0\hbar \omega_{0}$. The elastic current $I_{e}^{\mathrm{el}}$ as a function of μ and *U* for (**e**) $\lambda =0.01\hbar \omega_{0}$ (**f**) $\lambda =0.25\hbar \omega_{0}$. The other parameters were $\Delta \mu =0.01\hbar \omega_{0}$, $E_{l}=1.0\hbar \omega_{0}$, $E_{r}=2.0\hbar \omega_{0}$.

Figure 2 shows the elastic current $I_{e}^{el}$ as a function of λ, μ, and *U*. We can see that the there were two resonance peaks: A large λ excited more photons and enabled multi-photon-assisted tunneling. To better explain where the resonance peaks appeared, we plotted the imaginary part of the non-perturbative hybridized dot Green’s function $G_{0,\sigma }^{r}$ and the transmission function of the elastic current $T_{el}\left(\omega \right)$ as a function of ω.

**Figure 3:**
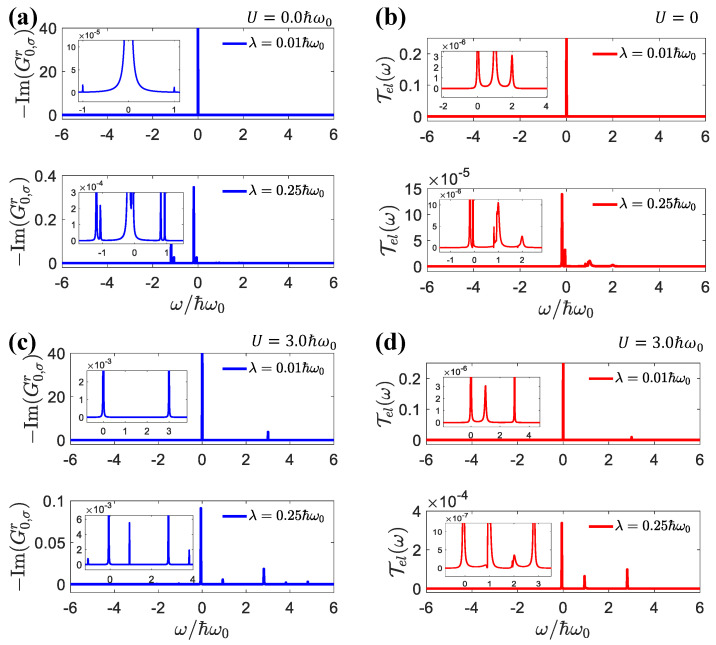
(**a**) The imaginary part of the retarded Green’s function $G_{0,\sigma }^{r}$, (**b**) transmission function of elastic current $T_{el}$ as a function of ω for U=0. (**c**) The imaginary part of the retarded Green’s function $G_{0,\sigma }^{r}$, (**d**) transmission function of elastic current $T_{el}$ as a function of ω for $U=3.0\hbar \omega_{0}$. The parameters were: $\Delta \mu =0.01\hbar \omega_{0}$, μ=0, $E_{l}=1.0\hbar \omega_{0}$, $E_{r}=2.0\hbar \omega_{0}$.

According to Equations (7) and (8), we could approximate −∂f/∂ω≈δ(ω−μ) at a low temperature. Then, the positions of the resonance peaks for Im(G0,σr) and $T_{el}\left(\omega \right)$ were given by $\mu =\Delta_{mn}^{\left(1\right)}=-\lambda^{2}$ and $\mu =\Delta_{mn}^{\left(2\right)}=-3\lambda^{2}+U$, respectively, for m=n (corresponding to the main peak in the density of states), where the resonance was strongest. As shown in Figure 3a,b, when the electron–electron interaction was turned off, i.e., U=0, we obtained ${\tilde{\varepsilon }}_{\sigma \bar{\sigma }}+U<{\tilde{\varepsilon }}_{\sigma }<0$. From Equation (8), the resonant peaks were then dominated by the $\Delta_{nm}^{\left(2\right)}<0$ contributions. When $\lambda =0.01\hbar \omega_{0}$, $\Delta_{mn}^{\left(1\right)}=\Delta_{mn}^{\left(2\right)}\approx 0$, and both the resonant peaks were at ω=0. However, for $\lambda =0.25\hbar \omega_{0}$, the two resonant peaks appeared at $\omega =-0.0625\hbar \omega_{0}$ and $\omega =-0.19\hbar \omega_{0}$, respectively. The strongest side peak appeared for n−m=1, with resonances at $\Delta_{mn}^{\left(1\right)}=\hbar \omega_{0}-\lambda^{2}$ and $\Delta_{mn}^{\left(2\right)}=\hbar \omega_{0}-3\lambda^{2}+U$, which was consistent with Figure 2a.

While $U=3.0\hbar \omega_{0}$ led to ${\tilde{\varepsilon }}_{\sigma \bar{\sigma }}+U>{\tilde{\varepsilon }}_{\sigma }$, there were two resonance branches, $\Delta_{nm}^{\left(2\right)}>0$ and $\Delta_{nm}^{\left(1\right)}<0$, as shown in Figure 3c,d. As shown in Figure 2a,b, for both weak and strong electron–electron interactions, with an increase in electron–photon interactions, we found that the peak value of the main branch decreased, but the peak value of the secondary branch increased. Similar results were found in [73], due to the reduction in the main peak and increase in the side peaks in the density of states when the light–matter interactions increased.

For the chemical potential μ>0, there was only one resonance, realized under the conditions of $\mu =-3\lambda^{2}+U$. Therefore, in Figure 2c,d, we observed that there was a strong resonance peak whose position was at $U=1.0\hbar \omega_{0}$ and $U=2.0\hbar \omega_{0}$ when λ was small, for the cases with $\mu =1.0\hbar \omega_{0}$ and $2.0\hbar \omega_{0}$, respectively. When $\lambda =0.25\hbar \omega_{0}$, the resonant values of *U* were increased to $1.1875\hbar \omega_{0}$ and $2.1875\hbar \omega_{0}$, respectively, by the strong light–matter interactions.

The resonant transport conditions are further revealed in Figure 2e,f. When the light–matter interactions were weak, $\lambda =0.01\hbar \omega_{0}$, and the resonance peaks were given by the single electron channel with $\mu =\varepsilon_{\sigma }\equiv 0$ and the double electron channel with $\mu =\varepsilon_{\sigma }+U=U$. For strong light–matter interactions, the resonance transport conditions were replicated as $\mu =-\lambda^{2}+\left(n-m\right)\hbar \omega_{0}$ for the single electron channel and $\mu =-3\lambda^{2}+U+\left(n-m\right)\hbar \omega_{0}$ for the double electron channel, with n≠m. Furthermore, for these two channels, the resonance chemical potentials were red-shifted by the $-\lambda^{2}$ and $-3\lambda^{2}$ terms, respectively.

**Figure 4:**
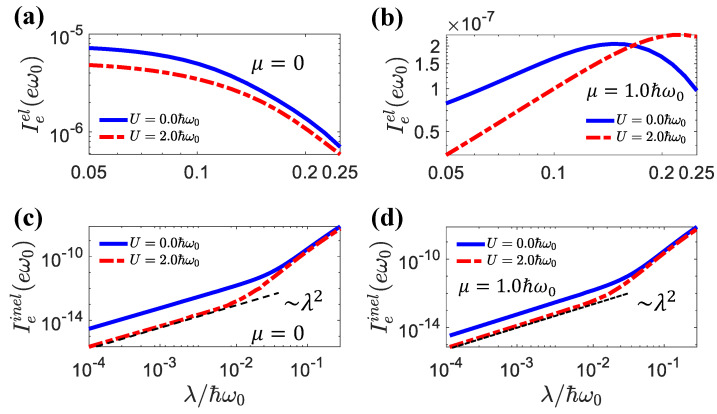
The elastic current $I_{e}^{\mathrm{el}}$ and inelastic current $I_{e}^{\mathrm{inel}}$ as a function of λ for different *U* values, (**a**,**c**) μ=0, (**b**,**d**) $\mu =1.0\hbar \omega_{0}$. The other parameters were $\Delta \mu =0.01\hbar \omega_{0}$, $E_{l}=1.0\hbar \omega_{0}$, $E_{r}=2.0\hbar \omega_{0}$.

In Figure 4, we present the elastic and inelastic currents as functions of λ. We can see that the inelastic processes induced by electron hopping with the assistance of photons were proportional to $\lambda^{2}$ when λ was small. This was consistent with the conventional physical picture presented by the Fermi–Golden rules. The dependence on λ became stronger when λ>0.0178, i.e., at the crossover between the weak-coupling and strong-coupling regimes. In contrast, the elastic current had a much weaker dependence on the light–matter coupling, since elastic transport does not require light–matter interaction. The latter affected the elastic transport indirectly by modifying the density of states via the sideband effects and the renormalization of polaron energy.

## 5. Rectification Effects

The principal ingredient for rectification is that the electronic system is asymmetric under forward and backward thermodynamic biases. However, if we introduced such asymmetry by shifting the electronic state in a distinct manner at positive and negative biases, the transport mechanism itself would rely on the inelastic interaction [78,79,80]. From Equation (26), one can see that the elastic transport was always anti-symmetric for forward and backward biases, even when the light–matter and electron–electron interactions were introduced. Therefore, the rectification effects came purely from inelastic transport.

The magnitude of the rectification effects was calibrated by [81,82,83,84,85,86]

(30) $$R_{e}=\frac{I_{e}\left(\Delta \mu \right)+I_{e}\left(-\Delta \mu \right)}{|I_{e}\left(\Delta \mu \right)|+|I_{e}\left(-\Delta \mu \right)|},$$

for the charge rectification, and

(31) $$R_{te}=\frac{I_{Q}\left(\Delta \mu \right)+I_{Q}\left(-\Delta \mu \right)}{|I_{Q}\left(\Delta \mu \right)|+|I_{Q}\left(-\Delta \mu \right)|},$$

for the Peltier rectification.

**Figure 5:**
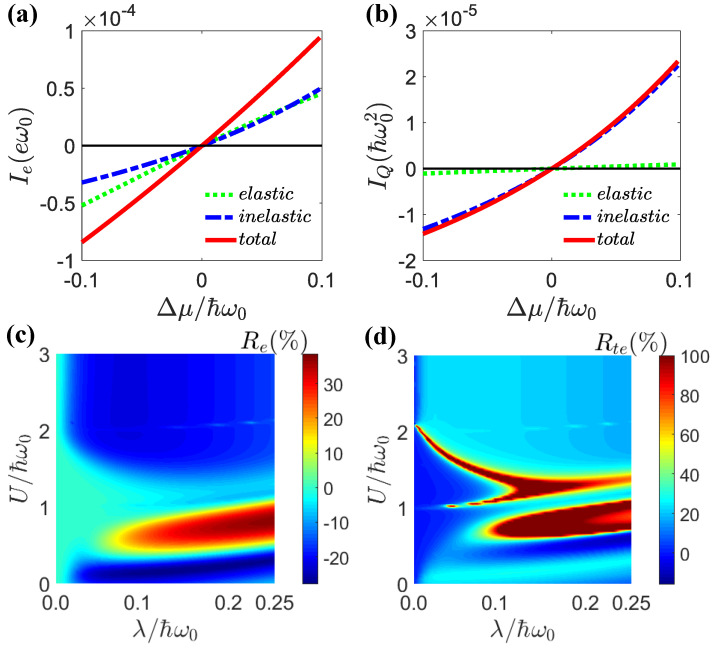
The charge current $I_{e}$ (**a**) and the heat current $I_{Q}$ (**b**) as a function of Δμ. The parameters were μ=0, $\lambda =0.1\hbar \omega_{0}$, U=0, $E_{l}=1.0\hbar \omega_{0}$, $E_{r}=2.0\hbar \omega_{0}$. (**c**) Charge rectification $R_{e}$ and (**d**) Peltier rectification $R_{te}$ as a function of λ and *U* for $\Delta \mu =0.1\hbar \omega_{0}$, $\mu =1.0\hbar \omega_{0}$. The other parameters were the same as in (**a**,**b**).

In Figure 5, we demonstrate the charge and Peltier rectification effects. The elastic and inelastic currents as functions of the voltage bias Δμ are shown in Figure 5a,b. From these figures, we observed that the elastic currents were anti-symmetric with respect to the forward and backward biases. Only the inelastic currents led to transport asymmetry and rectification effects. In this work, we mainly emphasize the effect of electron–electron interactions on rectification. The charge and Peltier rectification effects as functions of the light–matter interactions λ and electron–electron interactions *U* are presented Figure 5c,d. In general, strong light–matter and electron–electron interactions led to strong rectification effects.

**Figure 6:**
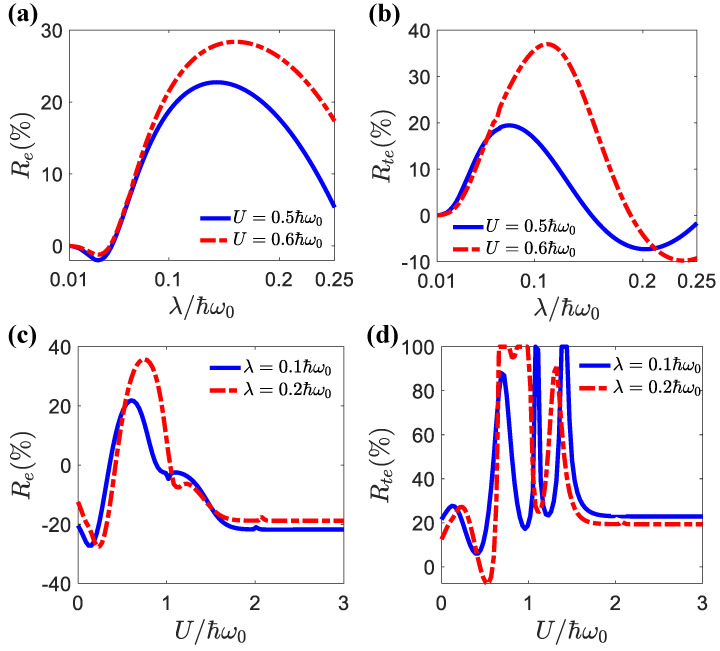
(**a**) $R_{e}$ (**b**) $R_{te}$ as a function of λ for different *U* values. (**c**) $R_{e}$ and (**d**) $R_{te}$ as a function of *U* for different λ values. The parameters were $\mu =1.0\hbar \omega_{0}$, $\Delta \mu =0.1\hbar \omega_{0}$, $E_{l}=0$, $E_{r}=1.0\hbar \omega_{0}$.

**Figure 7:**
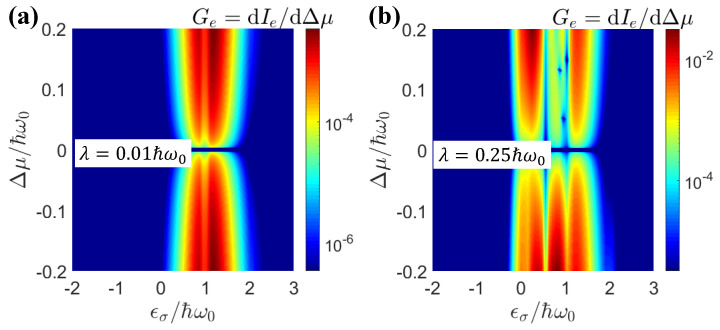
The differential electronic conductance $G_{e}=dI_{e}/d$Δμ as a functions quantum-dot energy $\varepsilon_{\sigma }$ and voltage bias Δμ for (**a**) $\lambda =0.01\hbar \omega_{0}$ (**b**) $\lambda =0.25\hbar \omega_{0}$. The parameters were $\mu =1.0\hbar \omega_{0}$, U=0, $E_{l}=1.0\hbar \omega_{0}$, $E_{r}=2.0\hbar \omega_{0}$.

In Figure 6a,b, we show the dependence of the charge and Peltier rectifications on the light–matter coupling λ for different electron–electron interactions. One can see that the maximum rectification occurred around $\lambda =0.15\hbar \omega_{0}$. Before this maximal value, the rectification effect increased with the light–matter strength λ. After this maximum, the rectification effect was weakened for stronger light–matter interactions. Figure 6c,d show that the dependences of the charge and Peltier rectifications on the electron–electron interactions were much more dramatic and nonmonotonic. The charge rectification presented simpler behavior, because it only depended on the particle current. The Peltier rectification depended on both the particle current and the average energy. Both of them could vary strongly according to the electron–electron interactions. As we showed in Figure 2 and Figure 3, electron–electron-interaction-induced transport resonance could be realized via the sidebands, leading to variations in the energy of the transported electrons. Therefore, the dependence of Peltier rectifications on the electron–electron interactions could be much stronger. Nevertheless, the general trend was that pronounced rectifications took place in the region $U\in \left(0.3,1.7\right)\hbar \omega_{0}$. In Figure 7, we show the differential charge conductance, $G_{e}=dI_{e}/d$Δμ, as a function of the quantum-dot energy $\varepsilon_{\sigma }$ and voltage bias Δμ for the weak and strong light–matter interaction cases. The weak light–matter interaction case demonstrated symmetric differential charge conductance with respect to the voltage bias Δμ. Therefore, the charge rectification was negligible for weak light–matter interactions. In contrast, the strong light–matter interaction case showed asymmetric differential conductance with respect to positive and negative voltage biases. It was precisely because of this asymmetry that the phenomenon of the rectification effect occurred. In addition, multiple resonance peaks emerged in the strong light–matter interaction case due to the sideband effect.

## 6. Thermal Transistor Effects in the Linear Transport Regime

In [81], it was proposed that a thermal transistor can be realized in a linear-response regime if phonon-assisted transport is dominant. However, the rate equation method used in [81] was confined to a weak electron–phonon coupling regime. In the weak coupling regime, i.e., with a small λ, the heat current amplification α>1 could be achieved when $|E_{l}-\mu |>|E_{l}-E_{r}|$ in the linear transport regime, where $E_{l}$ and $E_{r}$ are the quantum-dot energies. In [76], we extended this theory from weak a coupling regime to a strong light–matter interaction regime for a double-QD setup wherein we ignored the electron–electron interactions. Here, we used the rigorous Green’s function method to realize the linear thermal transistor effect in the c-QED system for arbitrary electron–photon interaction and electron–electron interaction strengths.

If we consider pure thermal conduction (i.e., the electrochemical potential difference is set to zero), the linear thermal transport properties of the system are given by [87,88,89,90,91,92,93,94]:

(32) $$\begin{array}{c} \left(\begin{array}{c} I_{Q}^{P}\\ I_{Q}^{R} \end{array}\right)=\left(\begin{array}{cc} K_{PP} & K_{PR}\\ K_{RP} & K_{RR} \end{array}\right)\left(\begin{array}{c} T_{P}-T_{L}\\ T_{R}-T_{L} \end{array}\right), \end{array}$$

where $K_{PP}=\frac{\partial I_{Q}^{P}}{\partial T_{P}}$, $K_{PR}=\frac{\partial I_{Q}^{P}}{\partial T_{R}}$, $K_{RP}=\frac{\partial I_{Q}^{R}}{\partial T_{P}}$, and $K_{RR}=\frac{\partial I_{Q}^{R}}{\partial T_{R}}$ are in the limit $T_{L},T_{R},T_{P}\to T$. Based on the above, the heat current amplification factor is given by:

(33) $$\alpha =|\frac{\partial_{T_{P}}I_{Q}^{R}}{\partial_{T_{P}}I_{Q}^{P}}|=\frac{K_{RP}}{K_{PP}},$$

**Figure 8:**
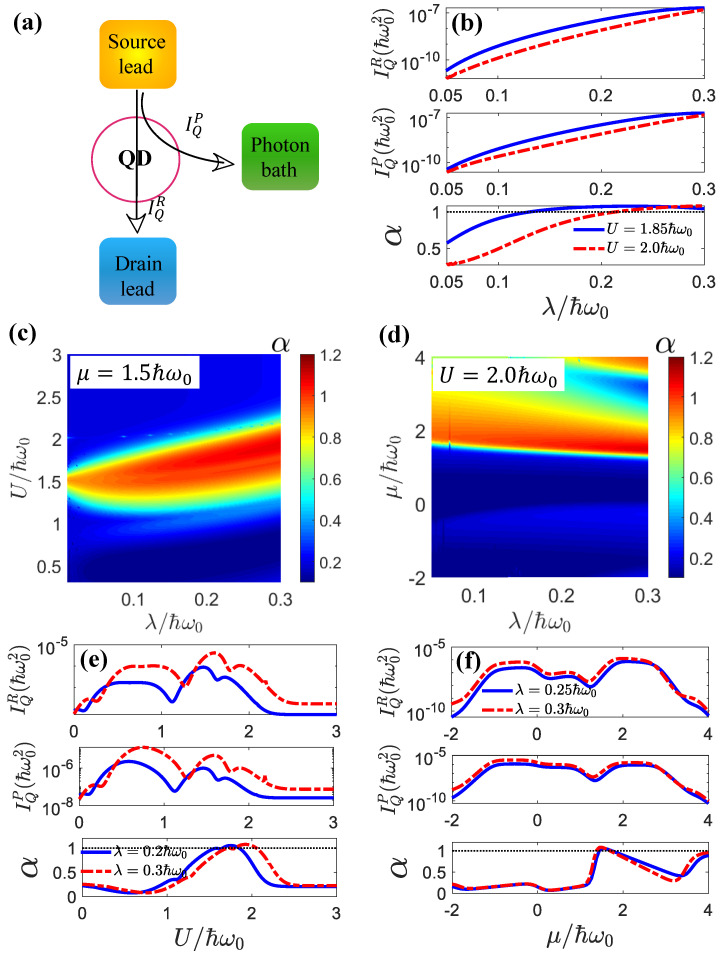
(**a**) Schematic of the c-QED system as a thermal transistor. The small heat current following from the source lead to the drain lead, $I_{Q}^{P}$, could control the large heat current following from the source lead to the gate lead (photon bath), $I_{Q}^{R}$. The ratio between the two heat currents defined the heat current amplification factor, α, which characterized the thermal transistor effect. (**b**) The heat current following the drain lead $I_{Q}^{R}$, the photonic heat current $I_{Q}^{P}$, and the amplification α as a function of λ for different *U* values. (**c**) The amplification factor α as a function of λ and *U* for $\mu =1.5\hbar \omega_{0}$. (**d**) The amplification factor α as a function of λ and μ for $U=2.0\hbar \omega_{0}$. (**e**) The heat current following the drain lead $I_{Q}^{R}$, the photonic heat current $I_{Q}^{P}$, and the amplification α as a function of *U* for different λ values, where $\mu =1.5\hbar \omega_{0}$. (**f**) The heat current following the drain lead $I_{Q}^{R}$, the photonic heat current $I_{Q}^{P}$, and the amplification α as a function of μ for different λ values, where $U=2.0\hbar \omega_{0}$. The other parameters were $E_{l}=1.0\hbar \omega_{0}$, $E_{r}=2.5\hbar \omega_{0}$.

In Figure 8a, we demonstrate precisely that the criterion for a transistor is α>1, which means that the current from the emitter to the collector is greater than the current from the emitter to the base. The source lead, drain, and photon bath served as the emitter, collector, and base, respectively. We showed that, via optimally engineering the parameters of the system, we could find regions where α>1. In our setup, the elastic electron-hopping mechanism with the assistance of photons from a photon bath was the main cause of the heat current amplification.

In order to hit hot spots of high α values, we plotted α as a function of λ for different *U* and μ values, as shown in Figure 8c,d. We found that the optimal regime for the transistor effect was $1.0\hbar \omega_{0}<U<3.0\hbar \omega_{0}$ and $1.0\hbar \omega_{0}<\mu <2.0\hbar \omega_{0}$. The strong coupling or even ultra-strong coupling regimes provided richer avenues for realizing heat current amplification. It was shown that a pronounced thermal transistor effect could be achieved for considerably large heat currents when the light–matter interactions and electron–electron interactions were strong (see Figure 8b,e,f). In general, strong light–matter interactions and electron–electron interactions helped the thermal transistor effect in the linear-transport regime. They also enhanced the heat currents significantly, since the photon heat current was proportional to the inelastic transition rate, which increased rapidly with the light–matter interactions, as shown in Figure 1. However, the dependences of the heat currents and the heat current amplification factor α on the electron–electron interactions *U* was quite nonmonotonic and showed complex behavior. These behaviors originated from the modulation of the sidebands via the electron–electron interactions *U*. The dependence of α on the chemical potential also reflected such modulation, which affected the resonant transport channel.

## 7. Conclusions and Outlook

In this work, we discussed a hybrid quantum impurity model with interacting electrons and photons. We demonstrated that one can achieve rectification and transistor effects by exploiting transport via photon-assisted hopping processes. By using the Keldysh diagrammatic NEGF method, we investigated the photonic and electronic currents of a QD c-QED system. The NEGF technique was well-suited to describing this hybrid quantum system. The method was valid for a wide spectrum of electron–photon interactions $\lambda <0.3\hbar \omega_{0}$ (going beyond the Fermi–Golden rule approach by including sidebands and polaron energy renormalizations) and arbitrary electron–electron interactions *U* when the transport was in the linear response regime or when the dot-lead couplings were much weaker than the electron–photon interactions.

By considering different observables, we demonstrated that the QD circuit QED system could serve as a robust diode and transistor, even when electron–electron interactions are taken into account. This effect is crucial for realizing photon-source quantum devices, as elucidated by our numerical investigation of the behavior of the nonequilibrium inelastic and elastic currents. We also showed that these systems exhibited thermal transistor effects even in the linear response regime by examining the heat current amplification factor. The nontrivial effects of the electron–electron interactions on the steady-state transport in both the linear and nonlinear regimes were revealed for a very wide range of interaction strengths, demonstrating complex and significant effects. Our findings may provide new platforms and opportunities for high-performance, high-energy-efficiency quantum thermoelectric devices.

Finally, it should be pointed out that our study was based on nonequilibrium steady-state transport, and the influence of the geometric effect [95,96,97,98] on periodically driven QD c-QED systems may be addressed in future studies.

## Data Availability

All data generated or analyzed during this study are available from the authors on reasonable request.
